# Stepwise Development of a Text Messaging-Based Bullying Prevention Program for Middle School Students (BullyDown)

**DOI:** 10.2196/mhealth.4936

**Published:** 2016-06-13

**Authors:** Michele L Ybarra, Tonya L Prescott, Dorothy L Espelage

**Affiliations:** ^1^ Center for Innovative Public Health Research San Clemente, CA United States; ^2^ College of Education University of Illinois at Urbana-Champaign Champaign, IL United States

**Keywords:** bullying, mhealth, text messaging, youth, prevention

## Abstract

**Background:**

Bullying is a significant public health issue among middle school-aged youth. Current prevention programs have only a moderate impact. Cell phone text messaging technology (mHealth) can potentially overcome existing challenges, particularly those that are structural (e.g., limited time that teachers can devote to non-educational topics). To date, the description of the development of empirically-based mHealth-delivered bullying prevention programs are lacking in the literature.

**Objective:**

To describe the development of BullyDown, a text messaging-based bullying prevention program for middle school students, guided by the Social-Emotional Learning model.

**Methods:**

We implemented five activities over a 12-month period: (1) national focus groups (n=37 youth) to gather acceptability of program components; (2) development of content; (3) a national Content Advisory Team (n=9 youth) to confirm content tone; and (4) an internal team test of software functionality followed by a beta test (n=22 youth) to confirm the enrollment protocol and the feasibility and acceptability of the program.

**Results:**

Recruitment experiences suggested that Facebook advertising was less efficient than using a recruitment firm to recruit youth nationally, and recruiting within schools for the pilot test was feasible. Feedback from the Content Advisory Team suggests a preference for 2-4 brief text messages per day. Beta test findings suggest that BullyDown is both feasible and acceptable: 100% of youth completed the follow-up survey, 86% of whom liked the program.

**Conclusions:**

Text messaging appears to be a feasible and acceptable delivery method for bullying prevention programming delivered to middle school students.

## Introduction

### Background

Bullying is a significant adolescent health issue: depending on the type of bullying, an estimated 9% to 38% of middle school students bully their peers sometimes or more often each semester [1]. Defined as intentional peer aggression that occurs repetitively, over time, between at least two people for whom differential power exists [2], bullying is associated with negative psychosocial correlates, including externalizing problems (eg, alcohol use) for bullies and internalizing behaviors (eg, depressive symptomatology) [3-5], and suicidal ideation and attempts for victims [5,6]. Emerging research suggests that bullying also has a negative physiological impact: victims have greater changes in C-reactive protein as they age into adulthood [7]. Research also suggests dysregulation of the hypothalamic-pituitary-adrenal axis among adults who were victims of bullying, resulting in memory deficits [8]. Recent work further notes shorter telomeres, which implicates a shorter life expectancy, among those exposed to violence (eg, bullying victimization) as children compared with nonexposed youth [9].

### The Current State of Bullying Prevention Programs

Existing bullying prevention programs seem to be having a modest impact. [10-16]. Existing approaches, which rely on teachers to deliver proscriptive content, may cause youth to dismiss the messages as a way of rejecting authority and exerting control over their social selves [16]. Programs that are able to remove the teacher from the content delivery may be able to overcome this challenge [17].

### The Potential for Technology to Affect Behavior Change

Many are exploring the potential for digital technology to enhance the educational experience and to promote prosocial behaviors [18,19]. The wide adoption of cell phones provides novel opportunities to “go where youth are.” Indeed, 88% of 12- to 17-year-old youth have access to a cell phone. Ninety percent of these youth use text messaging (short message service, SMS) interventions [20], which is the preferred mode of communication between peers [21]. Furthermore, text messaging is cost-effective: compared with the high personnel and infrastructure costs of in-person interventions, text messaging-based programs (ie, mHealth) are scalable and cost less than US$.02 per message to send and receive. Moreover, emerging evidence supports the efficacy of these programs [22-26].

### The Theory of Social-Emotional-Learning–Based Bullying Prevention Programs

Research suggests that effective behavior change programs are guided by strong theoretical models[27]. Social-Emotional- Learning (SEL)-based programs involve “the systematic development of a core set of social and emotional skills that help children more effectively handle life challenges and thrive in both their learning and their social environments” [28]. The model has emerged from influences across different movements that focused on resiliency and teaching social and emotional competencies to children and adolescents [29]. SEL is based on many well-established theories, including theories of emotional intelligence, social and emotional competence promotion, social developmental model, social information processing, and self-management [30]. It also integrates important aspects of several other behavior change models, including the health belief model, the theory of reasoned action, problem behavior theory, and social-cognitive theory [27,31].

SEL-based programs use social skills instruction to address behavior, discipline, safety, and academics in order to help youth become more self-aware, manage their emotions, build social skills (eg, empathy, perspective-taking, respect for diversity), build friendship skills, and decrease engagement in delinquent behavior [32-34]. SEL-based bullying prevention programs [34,35] target risk and protective factors that have consistently been associated with bullying and victimization in cross-sectional and longitudinal studies [36-43]. For example, empathy has been reliably found to be negatively associated with aggression and positively associated with prosocial skills [44-46]. The inverse correlation between aggression and empathy was found to be stronger in studies that focused on the emotional component of empathy than in studies in which cognitive empathy was measured [47]. Endresen and Olweus [47] conducted a study that specifically explored the association between empathy and bullying and brought attention to attitudes toward bullying, which have demonstrated positive correlations with bullying behavior [48]. Anger and hostility routinely emerge as important correlates of bullying. In several studies of bullying behavior, anger was the strongest predictor of bullying both cross-sectionally and longitudinally [48,49]. Impulsivity also plays an important role in bullying perpetration and victimization. In a prospective study of factors that predicted bullying over a 4-month period, Espelage et al [50] found a significant association between impulsivity and bullying behavior among a sample of 214 6th grade students. In addition, bullying prevention efforts have increasingly focused on the importance of bystander intervention, with significant results [37,51].

### A Description of BullyDown, a mHealth Bullying Prevention Program

**Figure 1:**
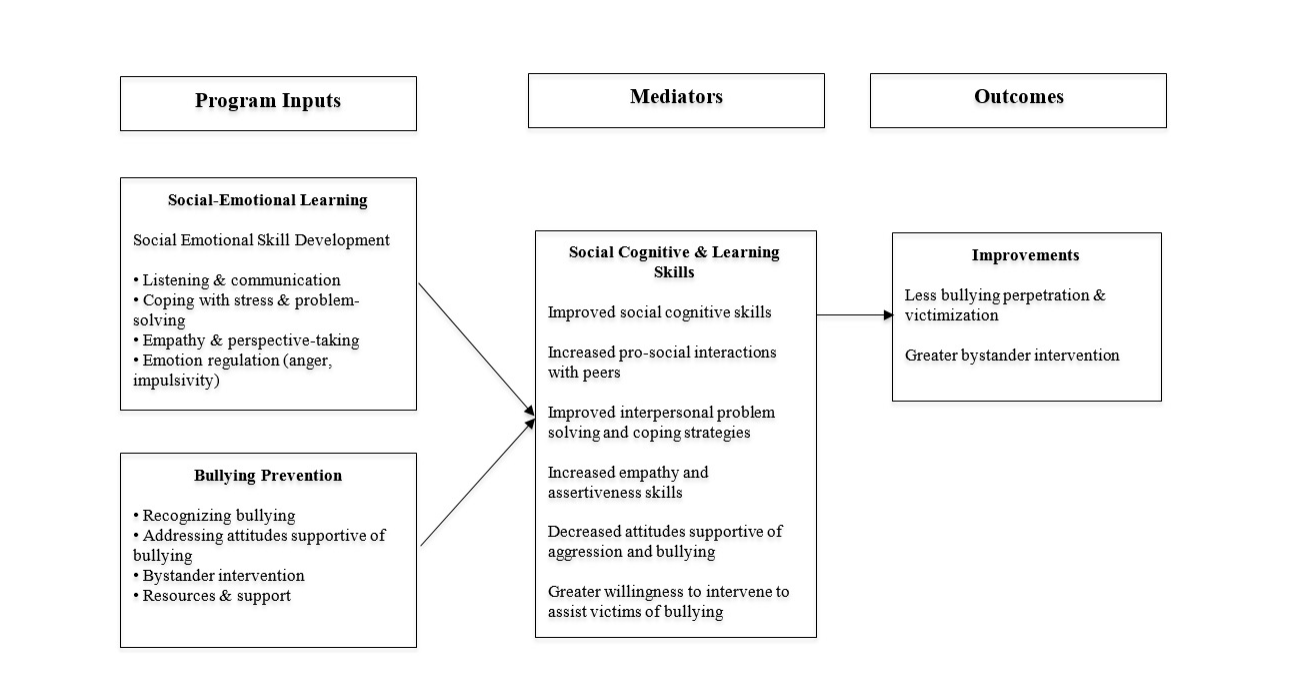
Social-Emotional Learning logic model.

SEL-based programs have reported positive results in terms of reducing bullying and other disruptive behaviors in middle school [34,52,53]. As such, we used the SEL model to guide the development of BullyDown, a mHealth bullying prevention program designed for middle school students (Figure 1).

According to the SEL model, interactivity increases participant engagement, and new behaviors need to be practiced to be integrated into one’s behavioral script. BullyDown therefore includes several features aimed at engaging youth, while also encouraging them to practice the skills discussed in the content. Principal among these is the “Text Buddy” feature, which pairs intervention participants as “buddies,” allowing them to discuss what they learn through the program with each other. Text Buddy has been used successfully in other text messaging–based programs and is associated with behavior change [54-56]. As such, we posit that the Text Buddies feature will provide an opportunity for youth to process the program information and to practice their new skills in a safe environment. To further integrate interactivity and also ensure that youth understand the program information, youth are given the opportunity to “level up” (ie, move to the next level) at the end of each week by answering a quiz question correctly.

A third interactive feature tested for acceptability is Happy Genie, which sends intervention participants on-demand messages that provide positive encouragement. This type of feature is specific to the target audience and the topic, but is based upon other behavior change programs that have also used on-demand features [54-56].

### Current Paper

Similar to iterative methods used in other mHealth programming [57,58], Ybarra and colleagues [56,59,60] have described a stepwise approach to developing and testing health behavior change content delivered via text messaging. Here, we describe the procedures and experiences encountered while developing BullyDown. Findings can inform the future design of similar programs for middle school youth, as well as those aimed at reducing bullying and other aggressive behaviors.

## Methods

The Chesapeake institutional review board reviewed and approved the research protocol. We sequentially implemented five activities: (1) online focus groups (FGs), (2) ongoing content development, (3) a Content Advisory Team (CAT), and (4) an internal team test, followed by (5) a beta test. We drafted content after conducting the FGs and continued refining content during the subsequent research activities. Research materials described herein are available via the Internet [61].

The development work spanned approximately 1 year: March 2014 to May 2015. For all research activities, study eligibility criteria matched those of the intended users of the intervention. Participants were enrolled in 7th or 8th grade (of exception, 6th graders took part in the FGs), owned a cell phone, were enrolled in an unlimited text messaging plan, intended to keep the same phone number for the next 6 months, had parental permission, and provided informed assent.

### Focus Groups Methods

**Figure 2:**
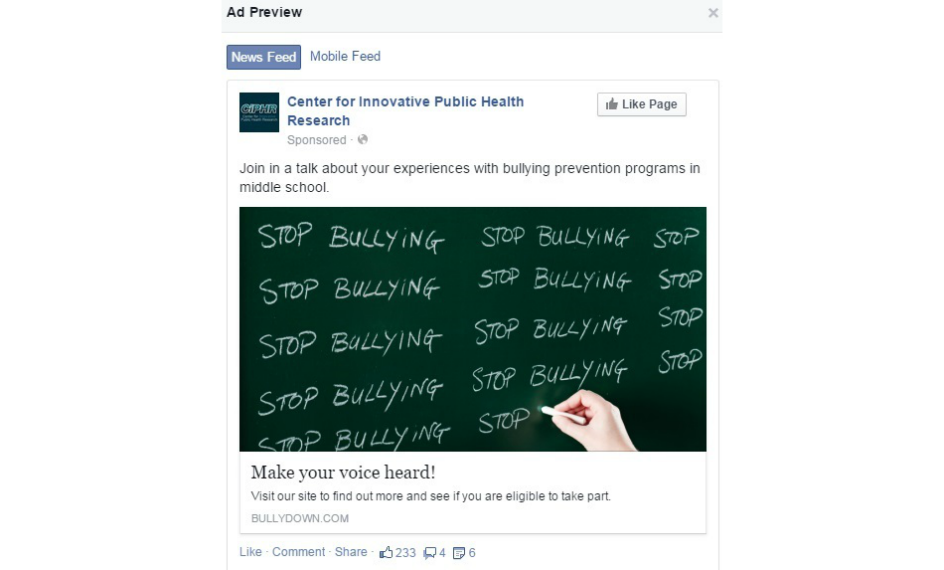
Focus group Facebook advertisements (2a).

The FGs aimed to: better understand how young people experience bullying and their prior exposure to bullying prevention programs in school, to obtain the “voice” of the target

population, and to query process issues. To gather feedback from a diverse group of youth nationally, our initial recruitment strategy relied on Facebook advertising targeted to US youth aged 13- to 14-years old (Figure 2).

Upon yielding a low response rate to the Facebook advertisements, we made subsequent changes to the recruitment strategy: increasing the Facebook ad budget from US$50 to US$100 per day, reducing the number of items on the Web-based screener to lower the completion time, and adding the company logo and link to the company website in the screener to increase the website legitimacy. We also contacted partner youth organizations who agreed to advertise for the research activity. When these attempts did not noticeably increase the number of eligible screeners, we contracted a research recruitment firm.

The research team developed a script of questions to guide the FG discussions. Example questions included:

When you think of the word “bullying,” what do you think of? How (if at all) is this different from what you think of when you think about “aggression?” What about being angry? Or getting into fights with friends?

Has someone asked you to think about what it might feel like to be bullied? What are things that you do that help you understand how others might be feeling? Like how it might feel to be bullied or to bully someone?

Additionally, the FGs queried process issues, including the preferred number and timing of daily text messages and feedback on possible intervention names.

Two FGs of 20 participants each were conducted. The script was written to span 3 days of questions. We posited that youths’ experiences and ability to articulate them would vary by grade level. As such, this was our stratification variable: FG1 was conducted with 6th and 7th graders, and FG2 with 7th and 8th graders. Participants logged into the bulletin board–style FG discussion on a password-protected site two to three times each day when and where it was convenient for them. Because of its asynchronous format, participants from across a wide geographic area could be included. Participants received a US$50 Amazon gift card as an incentive for complete participation during all 3 days.

**Figure 3:**
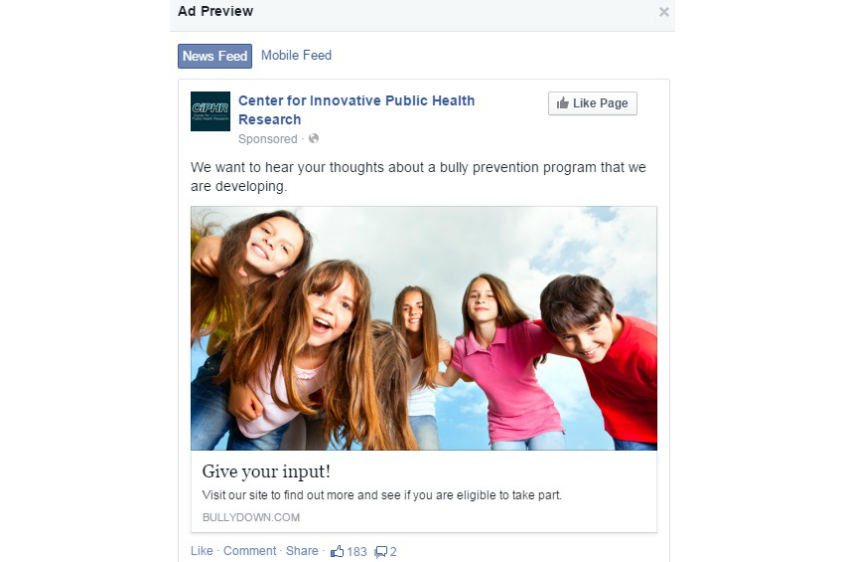
Focus group Facebook advertisements (2b).

### Content Advisory Team (CAT) Methods

We next convened a CAT to review and gather feedback about the saliency and understandability of the program content. The CAT also explored the acceptability of the three proposed program features: (1) Text Buddy, (2) Happy Genie, and (3) level-up questions.

To gather feedback, participants, or parents of participants when only the parent email address was available, received a Word document of the draft program text messages. The instructions asked youth to review and comment on each message within each week’s module (eg, Communication). Probes were included to help give direction about what they could consider when reading the proposed content (eg, Are you able to read it and understand it easily?). Instructions also emphasized that the messages were still being developed and also necessarily short given the character limit of text messages. However, during this stage, the proposed text messages were longer than 160 characters (e.g., 180 characters), as the aim was to first identify appropriate tone and content.

Next, participants took part in a 2-day online discussion to share feedback as a group. For each weekly topic, moderators asked youth to identify their favorite, least favorite, most helpful, and least helpful messages; any messages that felt too long or short; and what they learned from the module. Moderators also asked participants about the proposed program features and to suggest three of their own Happy Genie messages. Participants earned up to a US$50 amount in an Amazon gift card, US$30 for their individual review, and US$20 for their participation in the online discussion.

### Beta Test Methods

Findings were integrated into the program content, including feedback about the level-up questions and Happy Genie features. For example, to combat the judgment that bullies are “bad” (and therefore, implicitly, neither you nor your friends could be bullies) that emerged during the formative work, the level-up question during week 5 was:

Good morning and welcome to Week 5! Let's get you to Level 4. True or false: Bullies are mean to others because they are bad people. Text me back your answer.

Those who responded correctly received the following reinforcing message:

Bullies are mean for lots of reasons but that doesn't mean they are bad people. Maybe they just need help learning how to share their feelings. Hello, Level 4!

Those who responded incorrectly received the following response:

Actually, bullies can be mean for lots of reasons. That doesn't mean they are bad people. They might just need some help learning how to share their feelings.

Youth who answered incorrectly were given additional chances to answer a new question correctly before they were automatically moved on to the next level:

Let's try again. For Level 4: True or false: Young people who act “differently” are asking to be bullied.

Correct responses received the following:

Right! We're all different. Kids who act or look differently are just being themselves. They want to have friends just like everyone else. Hello, Level 4!

Incorrect responses resulted in this text:

We're all different. Teens who act or look differently are just being themselves. They want to have friends just like everyone else. Onward to Level 4!

Following a successful internal team test that confirmed the program software performed as intended and that the content was readable on cell phone screens, we conducted a beta test to confirm the feasibility of the protocol and technology in a school setting. To facilitate resolution of technology problems if needed, the beta test was conducted with participants from a low-income middle school in Illinois. Research staff went to the school in early December 2014 to screen 7th and 8th grade students from four preselected classrooms. Staff issued parental permission forms to eligible students to return in 1 week. During subsequent visits, research staff picked up the signed permission forms and facilitated enrollment (ie, obtaining informed assent from the students, helping the students complete the baseline Web-based survey in the school computer room, and verifying the students’ phones for compatibility with the software program).

Participants were randomized to either the 7.5-week intervention or control group at a 2:1 ratio. This maximized the amount of information gathered about the intervention, while also gathering sufficient data about the control group experience to determine its feasibility and acceptability. To avoid school hours, participants received one program message in the morning (7:15 AM) and the remainder of messages after school hours (between 4:00 and 9:00 PM). Intervention participants received three to six messages per day for a total of 214 messages, with an additional 15 to 29 messages from the level-up feature; were randomly assigned a Text Buddy; and had access to Happy Genie. Control participants received two messages weekly, one message encouraging thoughtful behavior (eg, A message from BullyDown: “Try doing one nice thing for someone today”) and the other thanking them for their participation, for a total of 14 messages. Control group participants did not have access to the Text Buddy and Happy Genie components.

In addition to the Web-based baseline survey, all participants were asked to complete a brief survey about their program experience, conducted via text message, every other week. We also conducted a Web-based follow-up survey 1 month post-intervention: youth received a link to the survey and two subsequent reminders via text message to facilitate self-completion. Those who did not complete the follow-up survey on their own could choose to complete the survey at school with research staff.

Participants received a US$10 cash incentive for returning the parent permission form, irrespective of whether they received permission. Participants received a US$25 Amazon gift card upon completion of the 1 month post-intervention follow-up survey.

## Results

Demographic characteristics of participants in the FGs and CAT, which were enrolled nationally; and the beta test, which was fielded in Illinois, are shown in Table 1. Bullying was somewhat common. For example, 23% (5/24) of beta test participants reported bullying someone via text messaging in the past 30 days.

### Focus Group Results

#### Recruitment and Enrollment

FG activities occurred between March and May 2014. Although the Facebook ads generated a high number of impressions (343,148) and unique clicks (2334) after 10 days of advertising, only 13 screeners were received. Of these, four were from eligible youth. Reasons for ineligibility included not owning a cell phone (7/13, 54%), not being enrolled in an unlimited text messaging plan (1/13, 8%), and being too young (1/13, 8%).

The recruitment firm identified 18 participants to take part in FG1 and 19 participants in FG2. Two participants from FG1 and one from FG2 assented and enrolled, but neither completed the FG nor provided a reason for their nonparticipation.

#### Process Issues

In total, the FG moderators posted 23 threads, each containing between one and five questions. As predicted, participant age was an important process variable: 6th grade youth had difficulty responding to questions. Homeschooled youth did as well.

For example, in response to familiarity with bullying prevention programming at school, a 12-year-old male said: “Im homeshooled so we don't have any of that.” (Note here, and throughout, that youth quotes are presented exactly as they were typed by participants.)

Participants overwhelmingly voted for the program name “BullyDown.” As one participant shared: “I like BullyDown.com the best. It states the purpose of the website and it's easy to remember!” (13-year-old female). Youth also expressed a preference for receiving two to four program text messages per day and felt that more would be too many.

#### Program Content

While some youth reported that their schools had not discussed bullying, most youth were able to articulate and also agreed with school definitions of bullying: “Our school considers any type of picking on or hurting someone's feelings bullying and I feel the same.” (12-year-old male). Overall, youth related to the definition of bullying as verbal, physical, and relational forms of aggression that can happen among friends and nonfriends:

Bullying comes in many forms. Physical, mental and cyber are all kinds of bullying. To me bullying is picking on or hurting someone you know can't defend themselves. A person bullies others to feel better or more important about themselves. Being aggressive towards another person is not the same as bullying. To me aggression is more about anger and the anger one feels towards another.

13-year-old female

Youth perceptions of victims varied, and in some cases, youth felt that some victims behaved in ways that contributed to their victimization. Youth also recognized that some students who are socially popular engage in bullying, making it more difficult to stand up to these bullies. Because emotion regulation is a core component of bullying prevention, we also asked FG participants about coping strategies they used when stressed or upset. Answers included: reading/writing, listening to music, counting, belly breathing, and talking to siblings and parents.

#### Integrating Focus Group Feedback into BullyDown

Based upon the SEL model, we drafted seven modules: (1) communication, (2) coping with stress and problem solving, (3) empathy, perspective-taking, and respect for diversity, (4) recognizing bullying, attitudes toward bullying, and attitudes supportive of aggression, (5) emotion regulation: anger, hostility, and impulsivity, (6) bystanders and intentions to intervene to help others, and (7) resources and support.

**Table 1:** Demographic characteristics of BullyDown participants by development activity.

Demographic characteristics		Focus groups (n=37) n (%)		CAT^a^ (n=9) n (%)		Beta test^b^ (n=22) n (%)	
Sex							
	Male	18 (48.7)		6 (66.7)		8 (36.4)
	Female	19 (51.4)		3 (33.3)		14 (63.6)
Age^c^							
	11 years	1 (2.7)		NA		NA
	12 years	17 (46.0)		4 (44.4)		10 (45.5)
	13 years	16 (43.2)		4 (44.4)		8 (36.4)
	14 years	3 (8.1)		1 (11.1)		4 (18.2)
Race/ethnicity^d^							
	Caucasian	18 (48.7)		6 (66.7)		10 (45.5)
	Black/African American	10 (27.0)		1 (11.1)		7 (31.8)
	Asian	1 (2.7)		0 (0.0)		0 (0.0)
	Mixed racial background	1 (2.7)		0 (0.0)		4 (18.2)
	Native American or Alaskan Native	0 (0.0)		1 (11.1)		0 (0.0)
	Hispanic	7 (18.9)		1 (11.1)		4 (18.2)
	Do not want to answer	0 (0.0)		0 (0.0)		1 (4.56)
Grade^c^							
	6th grade	12 (32.4)		NA		NA
	7th grade	14 (37.8)		4 (44.4)		11 (50.0)
	8th grade	11 (29.7)		5 (55.6)		11 (50.0)
Region							
	Northeast	9 (24.3)		2 (22.2)		0 (0.0)
	South	12 (32.4)		3 (33.3)		0 (0.0)
	Midwest	8 (21.6)		2 (22.2)		22 (100.0)
	West	8 (21.6)		2 (22.2)		0 (0.0)
Type of residence							
	Urban	12 (32.4)		3 (33.3)		Not asked
	Suburban	15 (40.5)		3 (33.3)		Not asked
	Rural	10 (27.0)		3 (33.3)		Not asked
	Been bullied^e^	10 (27.0)		6 (66.7)		14 (63.6)
	Bullied someone^e^	5 (13.5)		2 (22.2)		5 (22.7)

^a^Content Advisory Team.

^b^Beta test participants were enrolled from a partner school in Illinois, as such all participants are from the Midwest region.

^c^Grade eligibility criterion was modified prior to the CAT to include grades 7 and 8 only.

^d^Hispanic ethnicity was queried as part of race for the focus groups and CAT, and as a separate identity in the beta test. As such, race/ethnicity sums to more than 22 for the latter activity.

^e^For the focus groups and CAT, youth were asked if they had ever bullied and if they had ever been bullied. In the beta test, a more complex series of questions were asked, including different ways in which youth could be bullied, the mode through which they could be bullied (eg, in-person, via the Internet), and the place that they could be bullied (eg, at school, on the way to and from school). The timeframe was limited to the past 30 days.

**Table 2:** Example BullyDown program messages by SEL component.

SEL Component		BullyDown program text message	
Social			
	Communication		Passive communication is when you don't really share an opinion: “I don't care;” “Whatever you want is fine;” and things like that. *(Week 1)*
	Communication		It is also passive if you don't say anything. If someone teases you and you don't say anything, people may think you are ok with it or that it doesn’t upset you. *(Week 1)*
	Problem-solving		Step 1 in problem-solving is figuring out what the problem is. While this may sound easy, sometimes it can be harder than we think. *(Week 2)*
	Respect for diversity		We're all different. Teens who act or look differently are just being themselves. They want to have friends just like everyone else. *(Week 5)*
	Recognizing bullying		It can be a fine line between bullying and messing around. So how do you know where the line is? *(Week 4)*
	Recognizing bullying		One teen said that it is no longer drama, but becomes bullying when the other person doesn't fight back - or they fight back but it doesn't work. *(Week 4)*
	Attitudes supportive of bullying and aggression		Peer pressure is hard: maybe your friends dare you; or you think your friends will like you better; or your friends are teasing someone and you join in. *(Week 4)*
	Bystanders and intentions to intervene		Good news! You don't have to do it alone. Let's talk about ways you can help kids who are in trouble. *(Week 7)*
Emotional			
	Coping with stress		Let’s talk more about what to do when you’re down. Ask for help. Staying quiet doesn't make you stronger, often times it just makes you feel alone. *(Week 6)*
	Empathy and perspective taking		It can be easy to make bad decisions when we're angry. This is really true if we don't try to understand what we're feeling and why. *(Week 4)*
	Emotion regulation: anger, hostility		It's okay to get angry - we all get angry sometimes. It's what you do with that anger that is good or bad. *(Week 4)*
	Emotion regulation: impulsivity		Listening to music, writing about it, or talking to someone can help. Even just sleeping on it can help you see that it is not such a big deal the next day. *(Week 4)*

We incorporated quotes, as well as personal experiences shared by youth, from the FGs into program messages. For example, youth responses included:

Bullying is something done to someone weaker then you that you know cant defend themselves. When you go behind the other persons back it usally means your scared to say it to there face.

14-year-old male

I saw a boy making fun of a handicapped student right in front of a teacher and they did not say anything, so I did and then they stopped it and the boy who was bullying was sent to the office.

13-year-old female

These became adapted into the respective program messages:

Bullying can even be spreading a rumor about someone (true or not). It can happen face-to-face or behind your back. It can be online or through text messaging.

Another teen said: A boy was making fun of a handicapped teen in front of a teacher, but she didn't say anything. So I did. The boy was sent to the office.

In response to a question about how bullying might make a person feel, a participant shared:

it makes you feel badly about yourself and that feeling lasts for a long time. It doesn't go away quickly, even if others try to tell you it's not true. Deep inside you feel like it is true what they are saying. It stays with you and keeps coming back and being in your mind.

12-year-old female

This was translated into the following program message:

A girl who was bullied told me: It makes you feel badly about yourself and that feeling lasts for a long time. It doesn't go away quickly……even if others try to tell you it is not true. Deep inside you feel like what they’re saying is true. It stays with you and keeps coming back into your mind.

### Content Advisory Team Results

#### Recruitment and Retention

We conducted the CAT between August and September 2014. Given the success using a recruitment firm during the FGs, we determined this strategy to be the most efficient for subsequent research activities. We excluded 6th graders and homeschooled youth in these subsequent activities because of their difficulty responding to questions during the FGs. The recruitment firm recruited 12 youth for the CAT (Table 1). Fifty percent (6/12) of participants completed both the individual text message review and the online discussion. Twenty-five percent (3/12) of participants completed only the individual review. Of these three nonparticipants, one parent withdrew their child from the study due to illness and lack of time to complete the CAT. Another parent requested a Web-based version of the survey, which was provided although feedback was not received. The reason for the third youth’s nonparticipation is unknown.

Engagement with the CAT was low in part because of participants’ confusion about the protocol. Both the recruitment firm and the research staff received multiple requests from parents for clarification about the process. This confusion seemed to persist even after research staff explained the process to parents over the phone. Following up with participants and/or their parents was also difficult because messages from research staff often went unanswered. In many cases, the recruitment firm needed to contact parents and/or participants on behalf of research staff to resolve the issue. Response rates may also have been low because the CAT field period overlapped with the start of a new school year. After yielding little feedback, we extended the 7-day deadline by a week. To help invigorate completion, research staff provided participants with examples of the type of feedback that would be informative to program development. Interestingly, two participants requested that the messages be made available via the Internet through Google Docs. One did not have access to Word, and the other requested it for anticipated convenience.

Atypical of our past experience with adolescents [62], parents of CAT participants were often the point-of-contact. This is perhaps because the recruitment firm identified youth through their parents. As a result, we often lacked contact information for the youth. To avoid this issue in the future, researchers should consider including a study eligibility criterion that requires youth contact information or partnering with a recruitment firm that allows the project staff to talk directly to both parent and child at the outset of the study. Researchers should also be mindful of major transition periods for the target audience (eg, the beginning of a school year) when identifying desired field dates.

#### Text Message Review

As shown in Table 3, youth feedback to each of the messages were generally positive (eg, “Great job describing it”) and, in some cases, elicited personalized responses (eg, “I hate being accused of being bully”). Although some program messages received more negative feedback than others (eg, the message for recognizing bullying; emotion regulation: impulsivity; a quote from cartoon character BMO), at least one-half of the participants positively endorsed most messages. A participant also made a comment in the “emotion regulation: anger, hostility” section suggesting that some of the daily transitions between topics needed improvement.

#### Online Discussion

During the 2-day online group discussion, participants generally rated the program modules positively: between 80% (4/5) and 100% (5/5 or 4/4, depending on the number of youth who responded to the poll) of youth liked/strongly liked them. As an exception, 75% (3/4) of youth disliked the problem-solving module. Only one youth provided details on his negative reaction to the module:

I liked the how to deal with stress messages, the tips on what to do. The examples about starting rumors was nothing I would ever do, so did not like that.

12-year-old male

When asked about their overall impression of the program, many participants responded positively:

the program is perfect no changes needed.

13-year-old male

Less enthusiastic feedback was also received:

some of the messages were too long and boring and some of them were kinda confusing

12-year-old female

it was jumping from topic to topic.

13-year-old female

Four youth had negative reactions to the tone of some messages. For example, one youth shared:

[the messages] sounded like an adult trying to be ‘cool’ - the messages were too long and wordy and sounded like things my mom would say.

12-year-old male

When asked for further detail, youth specifically disliked the use of the phrase “rock star” in one of the messages. Although many participants liked the inclusion of quotes from other teens, one youth questioned the authenticity of the quotes (ie, emotion regulation: anger, hostility message; Table 3).

The level-up feature was well received, although it too was not immune to criticism. For example, some thought that while the feature was acceptable, the questions needed to be more challenging:

the questions to get to the next level were way too easy. the second try was even more easy. you should probably make it a little harder.

12-year-old male

Another participant thought that the questions felt like homework:

… I don't like having to 'earn' levels - it makes it more like a school assignment, not something that should be have to be done. why do you have to pass a level to move on? is there a prize or something to get through the levels. If I was doing this in real life and did not pass a level, I would just stop and not continue anymore.

12-year-old male

Regarding program features, participants were enthusiastic about Happy Genie:

This genie feature looks amazing this is just what is needed when their life feels like a bore and nothing is great about it. The feature that it sends them a positive quote is very nice indeed. Overall great feature really perked up my spirits with those quotes i am sure it will do just the same for the participants.

13-year-old male

This sentiment was echoed by a 12-year-old female:

I think this feature is great! It could be really helpful for a person whose having a bad day.

Youth also suggested several name alternatives, including Forever Friend, Good Day Genie, and the Happy Doctor.

**Table 3:** Example per-message feedback from the Content Advisory Team.

Message type	Specific program message	Participant feedback^a^			
		**12-year-old male**	**13-year-old female**	**12-year-old male**	**12-year-old male**
Communication	Assertive communication is when you’re clear about how you feel or what you want. Say someone is being mean to your friend. You could say: “I think the way you put him down was mean.”	*Ok, good*	*I like the messages when they describe an example of the type of communication which is what is happening in this one*	*Great job describing it*	*Glad itg explains it – but is too wordy*
Recognizing bullying	A teen told me that it's no longer drama, but becomes bullying when the other person doesn't fight back - or she fights back but it doesn't work.	*good*	*I like this message because as you know, I like hearing from other teens*	*Way to use examples*	*Not sure if that is right*
Attitudes supportive of bullying and aggression	Remember: There are lots of ways to make friends without bullying. If your friends are pressuring you to be mean to others, maybe they're not such good friends.	*Good message*	*I like this message because I think that so many people get caught up in who they want to be their friend, when they aren’t such good friends*	*Put a but before maybe*	*I stopped being friends with someone because I was tired of them bullying people all the time*
Coping with stress	There are lots of ways to deal with stress. You could go on a long walk, play basketball, read a book, go for a run or a bike ride, or even write a poem!	*Sounds good*	*I like this message because it has a variety of things to do when your stressed*	*Add “anything that helps*	*Good examples – but who would write a poem? Ick!*
Empathy and perspective taking	Maybe someone has called you a bully but you didn't know you hurt the person. This can be frustrating. Take a moment to think about why the other person may have felt bullied.	*yes*	*I like this message because I feel like if someone is accused of bullying, they seem to forget*	*I hate being accused of being bully*	*I have never been told I bullied someone*
Emotion regulation: anger, hostility	Imagine you’re in a fight with your friend. To deal with it, you could: stop talking to her. Shove her to show her you're mad. Make up a rumor about her. Or, tell her how you feel.	*good*	*I like this message but it is kind of a weird ending message for the day*	*They know what the right answer, but that would be awkward to say*	*Sometimes I would not talk to her but I would never shove her or start a rumor – that’s mean!*
Emotion regulation: anger, hostility	A teen told me: “I try to think about the situation before acting. If I can remember to do that, it works. But when I'm angry I usually forget those things.”	*ok*	*I like this message because I like hearing words from other teens*	*Way to put in a quote*	*No teen talks like that*
Emotion regulation: impulsivity	You don’t want to say something you’ll regret. Trying to get even with a bully continues the cycle. And it might put you at risk for something unsafe to happen.	*yes*	*I like this message because it is very clear and is easy to understand*	*Yea, the bully could bet you up*	*Not needed – bad message*
Quote	Great advice: When bad things happen, I know you want to believe they are a joke, but sometimes, life is scary and dark. That is why we must find the light.” - BMO)	*agree*	*I’m kind of confused with this and I’m not quite sure if this is supposed to be a quote or not*	*What is bmo?*	*Find the light? Not sure what this means*
		**13-year-old male**	**13-year-old female**	**12-year-old female**	**13-year-old male**
					
Communication	Assertive communication is when you’re clear about how you feel or what you want. Say someone is being mean to your friend. You could say: “I think the way you put him down was mean.”	*Great example on this type of communication.*	*i dont really use assertive communication*	*Needs a better example. Definition is clear*	*Bad*
Recognizing bullying	A teen told me that it's no longer drama, but becomes bullying when the other person doesn't fight back - or she fights back but it doesn't work.	*I don’t think this statement is true because I believe being able to identify it isn’t so simple*	*i dont agree*		*Ok*
Attitudes supportive of bullying and aggression	Remember: There are lots of ways to make friends without bullying. If your friends are pressuring you to be mean to others, maybe they're not such good friends.	*Another good comment and a way to point out that maybe someone who is pressuring you isn’t good to be around.*	*i hate it when my friend wants me to be mean. i hate being mean. i get a bad feeling inside*		*Ok*
Coping with stress	There are lots of ways to deal with stress. You could go on a long walk, play basketball, read a book, go for a run or a bike ride, or even write a poem!	*Great useful advice on how to deal with stress*	*i go outside*		*Ok*
Empathy and perspective taking	Maybe someone has called you a bully but you didn't know you hurt the person. This can be frustrating. Take a moment to think about why the other person may have felt bullied.	*I good suggestion and way to get someone to stop and think about their actions.*	*okay*		*Good*
Emotion regulation: anger, hostility	Imagine you’re in a fight with your friend. To deal with it, you could: stop talking to her. Shove her to show her you're mad. Make up a rumor about her. Or, tell her how you feel.	*This a great example for this type of situation.*	*id just tell her how i feel i guess*	*Make it a little more clear but good options*	*Good*
Emotion regulation: anger, hostility	A teen told me: “I try to think about the situation before acting. If I can remember to do that, it works. But when I'm angry I usually forget those things.”	*I like this comment I think it’s very true*	*i think sometimes before i act*		*Ok*
Emotion regulation: impulsivity	You don’t want to say something you’ll regret. Trying to get even with a bully continues the cycle. And it might put you at risk for something unsafe to happen.	*A good comment and way to look at it*	*getting even makes me feel bad even though they've hurt me*		*Feel like im being talked down to*
Quote	Great advice: When bad things happen, I know you want to believe they are a joke, but sometimes, life is scary and dark. That is why we must find the light.” - BMO)	*Very helpful advice and positive.*	*nice quote*		*Ok*

^a^To maintain participant voice, quotes are verbatim. As such, grammar and spelling errors exist.

#### Integrating Content Advisory Team Feedback Into BullyDown

Overlapping feedback provided by two or more youth were integrated into the program content where possible. For example, we deleted the word “rock star” and messages deemed particularly confusing (eg, the quote from BMO). We also modified the tone of messages to sound less motherly or adult-like. The message:

You can tell your friend how you feel. It might be hard. And it might not go well. But if you’re in a healthy friendship and you use assertive communication, it could go great!

Subsequently became:

Tell your friend how you feel. It might be hard, and it might not go well. But if you’re in a healthy friendship, and you talk assertively, it might go great!

We also reviewed the messages scheduled for the beginning and end of each program day for their flow and transitions, based on youth feedback.

We chose not to make the level-up questions more challenging as this seemed likely to make the feature further simulate homework. The level-up protocol was already designed to automatically move nonresponsive participants to the next module if they did not respond to the questions after a certain period. We established a plan to closely monitor participant reactions to the level-up feature during the beta test for further indication of acceptability.

We deemed participants’ positive review of Happy Genie as supportive of its acceptability. We renamed the feature “Forever Friend” based on the names suggested by participants and added inspirational messages (eg, A new day = a new beginning) and quotes from famous people (eg, “I’ve failed over and over again in my life. And that is why I succeed.” – Michael Jordan).

### Beta Test Results

#### Recruitment and Enrollment

Enrollment spanned 3 months and required research staff to visit the partnered middle school four times. Research staff screened 78 youth, 45% (35/78) of whom were eligible. Reasons for ineligibility included: not having a cell phone (21/78, 27%), not or uncertain if they were enrolled in an unlimited text messaging plan (9/78, 12%), and not planning to or uncertain if they would have the same phone number for at least 6 months (21/78, 27%), including the possibility that parents may take away phone privileges as a form of punishment.

Thirty-three eligible youth returned their permission forms, all of whom had parental permission to participate. Twenty-four eligible students provided written assent to participate, of which 22 completed the registration process and were randomized. Of the nine youth who had parental permission but did not enroll, three changed their mind, two moved, one was absent every time the research staff went to the school, and three had problems with phone access (eg, one shared the phone with a parent). Reasons for not completing the registration process included no longer being interested (n=1) and no longer having a cell phone (n=1). Participants were successfully randomized, resulting in 14 students assigned to BullyDown and eight to the control group (Table 1).

#### Process Issues

We conducted the beta test between December 2014 and May 2015. No concerns from the school administration or staff were expressed during the field period. Participants encountered some technology difficulties, however. The school’s firewall initially blocked the project enrollment website, but school technology staff quickly resolved the issue. Additionally, many students forgot to bring their phones to verify its compatibility with the program software. These students completed the Web-based baseline survey and were instructed to complete enrollment at home by responding with the word “verify” to the text message verification. Those randomized to the intervention group received a link to the Text Buddy Code of Conduct to read and accept. Some students had trouble completing the tasks, requiring research staff to return to the school to assist them in person.

Thirty-six percent (8/22) of participants completed the Web-based follow-up survey on their own time, and 14 completed it at school. The primary reason for not completing the survey independently was forgetting to do it. One participant thought the survey link did not look legitimate and so was hesitant to click on the link.

#### Program Acceptability and Feasibility

During the program delivery period, 86% (12/14) of intervention participants sent at least one message to their Text Buddy (range, 2-52), and 29% (4/14) sent at least one message to Happy Genie (range, 1-8). Half of intervention participants (7/14) responded to all seven weekly level-up questions, and 21% (3/14) responded to two or fewer level-up questions.

Feedback from the biweekly brief survey (Table 4) suggested that many intervention participants thought the messages were interesting and fun, although some felt the messages were boring (eg, week 2 messages). Seventy-five percent (9/12) of intervention youth were able to provide an example of a message they found memorable (week 4), and 75% (9/12) of youth provided a specific example of something they liked about the program (week 6).

**Table 4:** Beta test intervention group participant biweekly qualitative feedback.

Week	Program Message	Feedback per participant^a,b^			
		**13-year-old male**	**12-year-old male**	**13-year-old female**	**12-year-old female**
2	What do you think of the messages? Are they confusing? Boring? Interesting? Fun? Text me back what you think and why. The more details, the better.	*They are interesting and informative!*	*They have been helpful*	*Borin and fun, I already know half of the information you send me but I like the interactive texts*	*They are fun bc it tells me more information about the program*
	Is there anything about your experience in BullyDown that you'd like me to know at this point? Text your feedback or text “no.”	*I think there are too many texts per day.*	*Not right now no*	*No*	*U guys have told me most of the stuff I didn't know*
4	What one BullyDown text message sticks out in your mind, and why? This could be any message you've received since the start of the program.	*Being your loudest supporter because it shows that you should push yourself to do your best.*	*Believe you can and your halfway there.*	*All of the things to do when you are stressed..*	*To close ur eye when ur stressed*
	Is there anything about your experience in the program that you'd like me to know at this point? Text your feedback or text “no”.	*No*	*No*	*No.*	*No*
6	What is one thing that you really like about BullyDown and why? The more detailed you can be, the better.	*I like the daily advice and the inspirational messages.*	*I like all of the possible solutions. To the problems that kids have to deal with everyday. A Lot of. Kids don't kno how to handle situations*	*The one thing that I like is that it gives advice, it may be things that you already know it's something..*	*That I can learn more stuff about bullying and stuff like that I al so can ask questions about anything I need to know about bullying*
	And what is one thing that you really do not like about BullyDown or think that we need to make better, and why? Again, detail is helpful.	*Maybe not text as much through the day. Maybe just once in the morning and once at night.*	*Sometimes there are a lot of txts and it gets. Confusing*	*I think that BullyDown needs to be a place that people feel safe to explain how they feel and why they are feeling that way, but I honestly don't feel th at way. So to make it better you could let people talk with someone through text, instead of just giving advice.*	*I like everything about bully down*
	What else about your experience in the program would you like me to know at this point? Text your feedback or text “nothing”.	*Nothing*	*When I txt my bullydown friend. They don't reply*	*Nothing*	*Nothing*
8	How would you rate the number of messages that we send you each week? Too few, too many, or just right? Text me what you think.	*Too many.*	*Sometimes too many*	*Just right.*	*Just right*
	Is there anything about the program that you'd like me to know about your experience at this point? Text “no” or text your feedback.	*No*	*Having a bully buddy isn't a good idea. They don't reply to the text questions*	*No*	*No*
					
2	What do you think of the messages? Are they confusing? Boring? Interesting? Fun? Text me back what you think and why. The more details, the better.	*I am confused about what I should be texting to my text Buddy and when. I respond to your text when you ask a question but up until yesterday I hadnt texted my Buddy.*	*Interesting because I don't know what stuff you are going to text me.*	*Boring and interesting*	*Really idk its kinda confusing because when u asked me some of the question I got confused when I went to look for the answer and I got it wrong*
	Is there anything about your experience in BullyDown that you'd like me to know at this point? Text your feedback or text “no”.	*No*	*No*	*No*	*No not really*
4	What one BullyDown text message sticks out in your mind, and why? This could be any message you've received since the start of the program.	*When Im angry I usually go to my room and try to get something else on my mind. I end up later thinking about the situation and thinking it through.*	*The Forever Friend* ^c^ *texts. They cheer me up*	*The one about anger and the things I could do to prevent it. I liked that one because sometimes I get carried away and I get anger for no reason..*	*about calming your anger down why because it really helps when I am angry and when I am depressed about something*
	Is there anything about your experience in the program that you'd like me to know at this point? Text your feedback or text “no”.	*No*	*No.*	*No*	*No not really*
6	What is one thing that you really like about BullyDown and why? The more detailed you can be, the better.	*Ok*	*Forever friemd because it cheers me up*	*I like how y'all go in depth with every situation*	*ack and view what I have learned on here and what I already knew and if I was being a bully I would remember all the stuff I was taught*
	And what is one thing that you really do not like about BullyDown or think that we need to make better, and why? Again, detail is helpful.	*Sometimes the times of the texts are not convenient for a response since we are students that may be involved in other activities.*	*The texts I get back to back because I can't keep up*	*Maybe that y'all could slow down on the text messages*	*I like how they go step by step trying to prevent us from being and getting bullied I like that because if someone was gettin bullied I can either look b*
	What else about your experience in the program would you like me to know at this point? Text your feedback or text “nothing”.	*Ok*	*Nothing*	*Nothing*	*Nothing*
8	How would you rate the number of messages that we send you each week? Too few, too many, or just right? Text me what you think.	*Ok*	*Just right*	*Just right*	*Too many*
	Is there anything about the program that you'd like me to know about your experience at this point? Text “no” or text your feedback.	*No*	*No*	*No*	*No*
		**12-year-old female**	**13-year-old female**	**12-year-old female**	**13-year-old male**
					
2	What do you think of the messages? Are they confusing? Boring? Interesting? Fun? Text me back what you think and why. The more details, the better.	*Confusing Also I still havent talk to my buddy*	*Boring*		
	Is there anything about your experience in BullyDown that you'd like me to know at this point? Text your feedback or text “no”.				
4	What one BullyDown text message sticks out in your mind, and why? This could be any message you've received since the start of the program.	*Ok*		*The one about if my friend doesn't tell me she isn't coming to lunch*	
	Is there anything about your experience in the program that you'd like me to know at this point? Text your feedback or text “no”.	*Who is my buddy and how do I contact them*		*No*	
6	What is one thing that you really like about BullyDown and why? The more detailed you can be, the better.	*Yes*		*It. Gives you advice*	*I like Bullydown because it has really made me see how people can get bullied and how it can effect them and the different types of buling there is*
	And what is one thing that you really do not like about BullyDown or think that we need to make better, and why? Again, detail is helpful.	*Nothing*		*Make questions more deep*	*I really don't think that you men and women missed anything major*
	What else about your experience in the program would you like me to know at this point? Text your feedback or text “nothing”.	*Nonthing*		*Nothing*	
8	How would you rate the number of messages that we send you each week? Too few, too many, or just right? Text me what you think.				
	Is there anything about the program that you'd like me to know about your experience at this point? Text “no” or text your feedback.				

^a^To maintain participant voice, quotes are verbatim. As such, grammar and spelling errors exist.

^b^Blank spaces indicate a lack of response.

^c^“Forever Friend” refers to the Happy Genie feature.

Moreover, all participants said they would recommend the program to a friend when asked at the end of week 6 (Table 5). Opportunities for improvement were also noted: although 80% (8/10) of intervention participants agreed that reading the messages the same day they were received was easy (Table 5), five youth shared concerns about the message quantity or timing when asked at the end of week 6 about something they may not like about the program (Table 4).

Feedback from intervention participants suggested that they were enthusiastic about having a Text Buddy. This feature also drew at least one negative response each week, however (Table 4). Subsequent follow-up with youth revealed that their discomfort centered on being paired with someone from the same school and having to talk about bullying with a schoolmate: “…A Lot of kids in this area kno each other and don't want to talk about things like this.” (12-year-old male). Some youth also expressed frustration that their buddies were unresponsive to their messages; or felt discomfort about being paired with someone of the other sex or in a different grade.

As shown in Table 5, both the intervention and control group content appeared to be acceptable: more than four in five youth in each arm agreed that they liked the program. With 88% (7/8) of control participants saying that the program helped them not bully others in the future, this arm, although of lesser intensity than the intervention arm, appeared to blind participants to their arm assignment. In addition to high scores for understandability and salience of program content, the experience also seemed to be feasible: most youth disagreed or strongly disagreed that the program sent too many messages, and none of the beta test participants in either arm agreed that they stopped reading the messages by the end of the program.

**Table 5:** Acceptability and feasibility data from pilot test participants.

Youth responses			Control n (%)^a^		Intervention n (%)	
Biweekly survey in field						
			(n=6)		(n=10)	
	How much are you liking BullyDown? (Week 2)		NA^b,c^		8 (80.0)	
	How easy or hard has it been to read your texts the *same* day we send them to you? (Week 4)		5 (83.3)		8 (80.0)	
			(n=5)		(n=8)	
	How likely are you to recommend BullyDown to your friends? (Week 6)		3 (60.0)		8 (100.0)	
One-month follow-up survey			(n=8)		(n=14)	
Acceptability (Agree/Strongly Agree)						
	I like the program		7 (87.5)		12 (85.7)	
	I learned things in BullyDown…					
		That will help me not bully others in the future	7 (87.5)		14 (100.0)
		That will help me not be bullied by others in the future	6 (75.0)		12 (85.7)
		That will help me stop bullying when I see it happening to others	6 (75.0)		13 (92.9)
	I do not think people like me should go through the BullyDown program (Disagree/Strongly Disagree)		7 (87.5)		10 (71.4)	
	BullyDown talked too much about… (disagree/strongly disagree)					
		Feelings	7 (87.5)		8 (57.1)
		Bullying	7 (87.5)		11 (78.6)
	The text messages were easy to understand		6 (75.0)		12 (85.7)	
	BullyDown talked about things my friends and I experience in our lives		6 (75.0)		11 (78.6)	
Feasibility (disagree/strongly disagree)						
	BullyDown sent too many messages		7 (87.5)		10 (71.4)	
	I stopped reading the messages by the end of the BullyDown program		8 (100.0)		10 (71.4)	
	BullyDown got in the way of my daily schedule		8 (100.0)		10 (71.4)	

^a^Percentages reflect those in the extreme categories: agree or strongly agree (4 or 5 on a 5-point Likert scale; 7-10 on a 10-point ordinal scale); disagree or strongly disagree (1 or 2 on a 5-point Likert scale; 1 or 2 on a 5-point ordinal scale).

^b^The control group inadvertently did not receive the Week 2 biweekly survey.

^c^not asked.

The enrollment process seemed to require in-person facilitation by research staff at a level that is at least commensurate with an in-person intervention. Both interesting and potentially problematic, 33% (11/33) of youth who were eligible and had parental permission lost interest in participating before enrollment. Given the small sample size, this may be an anomaly. Further investigation of this feasibility issue in a larger trial is needed before conclusions can be drawn. Also, some participants shared that they would have been more likely to respond to the follow-up survey if the link had been sent by email instead of text message. Future efforts should consider collecting multiple forms of contact (eg, Facebook, email address).

Despite CAT participants’ expressed interest in the concept of Text Buddy, most beta test participants only sent one or two messages to their buddy. Based upon their feedback, future trials could ideally match Text Buddies who are attending different schools and, when possible, of the same sex and grade level. An interactive guide, either in-person or via the Internet, instructing how to use the feature could also be incorporated into the content. Additionally, only participants with expressed interest in the Text Buddy feature could be matched to reduce the likelihood of unresponsive buddies.

Several intervention youth expressed concern about the intensity and/or timing of the daily messages during the field period but not when it was assessed at 1-month follow-up. This feedback most likely arose on days that had five or six messages, resulting in participants receiving four or five messages across a 4-hour window after school. To ameliorate this, the total number of messages per day in future iterations of the program will be limited to four where possible.

Consistent with feedback from some CAT participants who thought the level-up questions were too easy, one beta test participant thought that the questions should be more meaningful. Given the lack of negative feedback from other beta test participants about the level-up experience and the desire to avoid simulating homework however, more feedback from randomized controlled trial participants will be solicited before making significant changes.

### Discussion

### Principle Findings

Stepwise development of BullyDown over a 1-year period helped ensure the program was generally well received by middle school students. Findings reveal important insights about developing mHealth interventions for younger youth, particularly those focused on bullying prevention, including the preferred number of messages per day (ie, 2-4 messages), a desire for shorter messages, and the ideal age range (ie, 7th grade and older). In interventions aimed at older adolescents (e.g. 14- to 17-years old), others have noted a similar preference among participants for a low-intensity experience in interventions [63,64]; however, higher intensity programs (eg, 8-15 messages per day) have also received indications of acceptability [56]. The desired threshold seems likely to be affected not only by the age of the youth but also the topic in question. This variability in participants’ acceptability of various program intensities highlights the importance of iteratively developing new mHealth program using ongoing feedback from the target population.

Also contrasting other mHealth adolescent recruitment efforts [65], Facebook was less efficient in nationally recruiting youth than was a more traditional recruitment method (ie, using a recruitment firm). Initial interest in the ads was commensurate with expectations for a successful campaign, but completion of the Web-based screener seemed to be an issue. Perhaps younger youth are more cautious when providing contact information to unknown people or organizations via the Internet, or perhaps the topic (ie, bullying prevention), when more fully explained, is of little interest to youth. However, the school administrator’s enthusiasm for the beta test suggests that the program is acceptable and feasible for implementation in school-based samples. It also supports a hypothesis that the eventual program will need to be promoted to school administrators rather than youth directly.

Several additional differences between the experiences in the BullyDown beta test with the implementation of other text messaging–based health behavior change programs should be noted. For example, compared with high school-aged youth [56], middle school students' relationship with their phones seems to be less predictable: (1) they do not carry their phones everywhere – at least not to school and not necessarily outside of school, (2) they might not remember their phone number accurately, and (3) their access appears to be more tenuous – parents are more likely to restrict their cell phone use. Also, staff resources needed to enroll youth appear to be commensurate with in-person interventions. This means that cost savings in enrollment will not be realized. Once the intervention program messages begin however, most 7th and 8th graders are able to engage with the program on their own, suggesting that once engaged, a standalone technology intervention has promise. These differences highlight the heterogeneity of mHealth research: simply because a particular protocol works for one program and population does not mean the same protocol will work with other programs and populations.

Although control and intervention participants attended classes side by side, the control arm appears to have been blinded: they were equally likely as the intervention arm to say they liked the program, and a similar percent of youth said they learned valuable things about bullying during the program. This suggests that randomization at the individual-level may not pose a serious threat to contamination; however, results should be replicated in a larger sample size.

### Limitations

Specific program recommendations that emerged from this work may not generalize to other health behavior change topics or youth populations. Although the national recruitment strategy is a strength, FG and CAT participants may not be representative of all youth given they were identified through a recruitment firm. Beta test participants were recruited from a disadvantaged public middle school in Illinois and may not be representative of students in other settings. More generally, youth who have a cell phone and are enrolled in an unlimited text messaging plan may be different from youth who do not meet these criteria. It seems unlikely however that youth who lack a cell phone or are not on an unlimited text messaging plan would opt into such a program if it was publicly available. Additionally, confirming that youth actually read the text messages is not possible beyond their self-report; therefore, actual exposure is unknown.

### Conclusion

Increasingly, SEL programs are being implemented in schools across the United States to address a wide-range of problematic behaviors (eg, bullying, delinquency) and to promote academic success. However, the majority of these programs include curricula that range from 15 to 20 or more lessons, requiring significant instructional time and resources that some school districts may not be able to provide. BullyDown harnesses the benefits of text messaging, such as being able to go where youth are and communicating important information in consumable chunks, to offer a bullying prevention program that can be delivered outside of school and without the need for facilitator time or resources. While expensive in time and money, iterative intervention refinement increases the likelihood that the resulting intervention is salient to the target population while retaining its adherence to theory. 

Given the positive feedback from beta test participants about the program content and the experience as well as the 100% (22/22) retention rate, the BullyDown mHealth bullying prevention program appears to be both feasible and acceptable for implementation in a middle school setting with 7th and 8th grade youth. Our next step will be to test the intervention in a large randomized controlled trial to see if exposure is associated with reduction in bullying behavior.

## Abbreviations

CAT: content advisory team
FG: focus group
SEL: social-emotional-learning
SMS: short message service
